# Does comorbidity increase the risk of patients with COVID-19: evidence from meta-analysis

**DOI:** 10.18632/aging.103000

**Published:** 2020-04-08

**Authors:** Bolin Wang, Ruobao Li, Zhong Lu, Yan Huang

**Affiliations:** 1 Weifang Medical University, Weifang 261031, China; 2 Department of Human Anatomy, Weifang Medical University, Weifang 261031, China; 3 Department of Oncology, Affiliated Hospital of Weifang Medical University, Weifang 261031, China

**Keywords:** COVID-19, comorbidity, meta-analysis, risk

## Abstract

Currently, the number of patients with coronavirus disease 2019 (COVID-19) has increased rapidly, but relationship between comorbidity and patients with COVID-19 still not clear. The aim was to explore whether the presence of common comorbidities increases COVID-19 patients’ risk. A literature search was performed using the electronic platforms (PubMed, Cochrane Library, Embase, and other databases) to obtain relevant research studies published up to March 1, 2020. Relevant data of research endpoints in each study were extracted and merged. All data analysis was performed using Stata12.0 software. A total of 1558 patients with COVID-19 in 6 studies were enrolled in our meta-analysis eventually. Hypertension (OR: 2.29, P<0.001), diabetes (OR: 2.47, P<0.001), chronic obstructive pulmonary disease (COPD) (OR: 5.97, P<0.001), cardiovascular disease (OR: 2.93, P<0.001), and cerebrovascular disease (OR:3.89, P=0.002)were independent risk factors associated with COVID-19 patients. The meta-analysis revealed no correlation between increased risk of COVID-19 and liver disease, malignancy, or renal disease. Hypertension, diabetes, COPD, cardiovascular disease, and cerebrovascular disease are major risk factors for patients with COVID-19. Knowledge of these risk factors can be a resource for clinicians in the early appropriate medical management of patients with COVID-19.

## INTRODUCTION

Coronavirus Disease 2019 (COVID-19) is a viral respiratory disease caused by the 2019 novel coronavirus (2019-nCoV), which has caused the pneumonia epidemic in the world [1–3]. As of March 5, 2020, a total of 96539 cases with laboratory-confirmed COVID-19 infection have been detected in the world reported by the World Health Organization (WHO). In China, there have been 80567 accumulated confirmed cases of COVID-19, and 5952 of them were existing severe patients. Given the rapid spread and high mortality rate of COVID-19, it is absolutely necessary to evaluate the possible risk factors affecting the progression of disease in COVID-19 patients.

Previous studies show that COVID-19 patients with comorbidity may lead to a poor prognosis [5]. Identifying the most important risk groups is essential when making decisions anti-2019-nCoV therapy. To date, there has been no systematic review that comprehensively explores whether the presence of common comorbidities increase COVID-19 patients’ risk, to guide clinical practice better. Therefore, we performed a meta-analysis of the available studies to explore relationship between comorbidity and patients with COVID-19.

## RESULTS

### Literature search and screening

**Figure 1:**
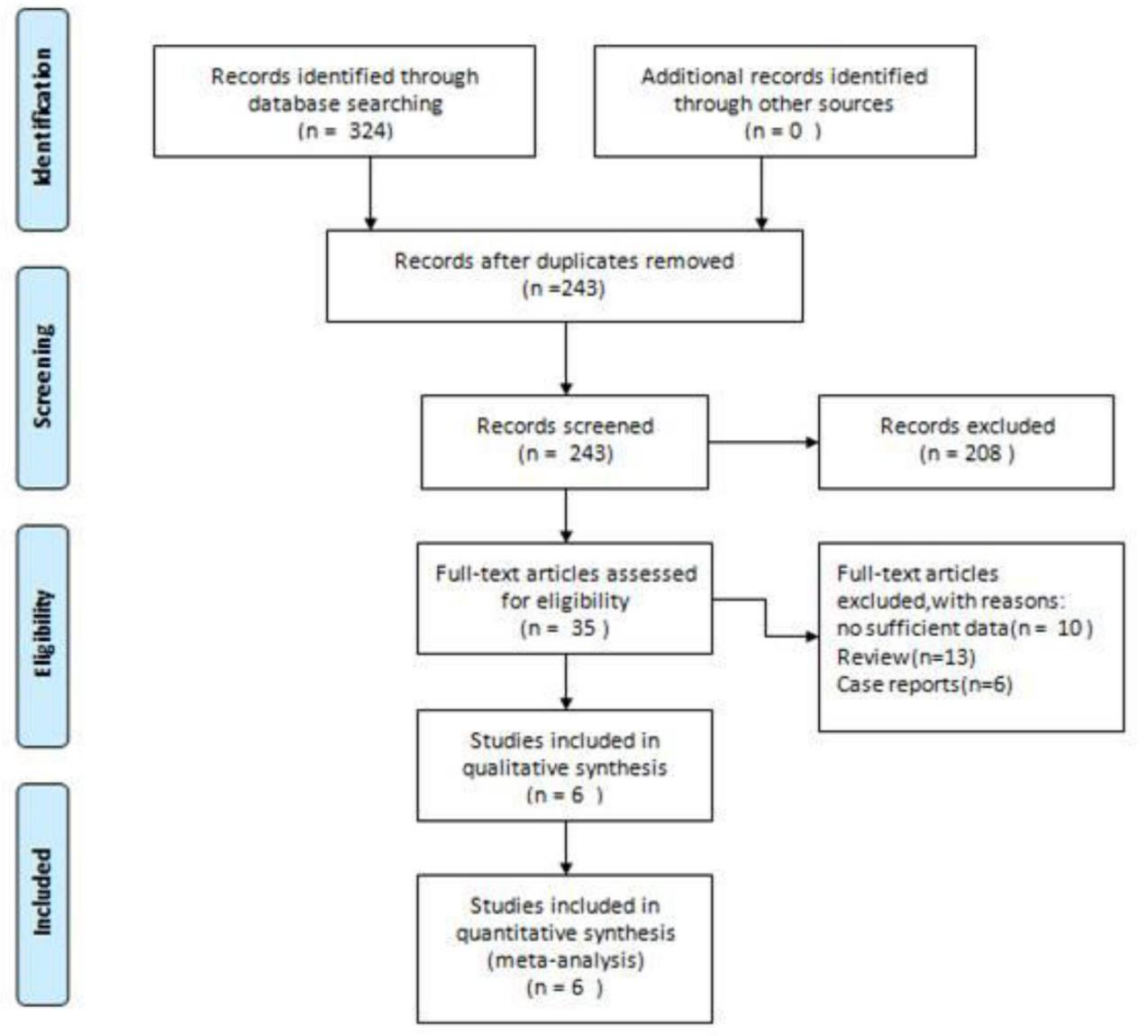
**Flow diagram of the literature search and selection process in the meta-analysis.**

The database searches identified a total of 324 potentially relevant articles. After the exclusion of duplicate references,243 articles were considered for the meta-analysis. Of these,208 studies were excluded after screening the title and abstract. After careful review of the full texts, 29 articles were excluded because they were reviews, cases, and insufficient data. Six studies qualified for inclusion [1, 4–8]. The flow diagram (Figure 1) showed the detailed literature search steps.

### Characteristics and quality of studies

A total of 1558 samples from 6 retrospective studies were enrolled in this meta-analysis [1, 4–8]. All six studies were performed in China. Six studies [1, 4–8] reported that hypertension, diabetes, and COPD, five covered liver disease [1, 4–7], four investigated malignancy [1, 4, 5, 8], renal disease [4–7], and cardiovascular disease [4–7], and three [4, 5, 7] researched cerebrovascular disease. Two studies [1, 5] used whether patients experienced ICU care to judge the severity of the disease, and the other four studies used clinical symptoms to judge the severity of the disease. All articles are of high quality because of NOS score no less than 6. Detailed descriptions of the studies included are shown in Table 1.

**Table 1:** Main characteristics of the included studies in our-analysis.

**Study**	**Year**	**Country**	**Sample**	**Median Age (years)**	**Sex**		**Diseases severity**		**Basis of disease severity**	**NOS**
					**Men**	**Women**	**Non-severe**	**Severe**		
C.Huang	2020	China	41	49.0 (41.0–58.0)	30	11	28	13	ICU care	7
D.Wang	2020	China	138	56.0 (42.0-68.0)	75	63	102	36	ICU care	7
W.Guan	2020	China	1099	47.0 (35.0–58.0)	640	459	926	173	clinical symptoms	8
W.Liu	2020	China	78	38.0 (33.0-57.0)	39	39	67	11	clinical symptoms	7
X.Xu	2020	China	62	41.0 (32.0-52.0)	36	26	29	33	clinical symptoms	6
J.Zhang	2020	China	140	57.0 (25.0-87.0)	71	69	82	58	clinical symptoms	7

NOS, Newcastle-Ottawa Scale.

### Hypertension, diabetes, and COPD

Six studies, including 324 severe group cases and 1234 non-severe group cases, provided the data in terms of hypertension, diabetes, and COPD [1, 4–8]. The heterogeneity test showed low heterogeneity among these studies, and a fixed-effects model was used for the meta-analysis. The results find that COVID-19 patients with hypertension (OR: 2.29, 95% CI: 1.69-3.10, P<0.001) (Figure 2A), diabetes (OR: 2.47, 95% CI: 1.67-3.66, P<0.001) (Figure 2B), or COPD (OR: 5.97, 95% CI: 2.49-14.29, P<0.001) (Figure 2C) had a higher risk of exacerbation.

**Figure 2:**
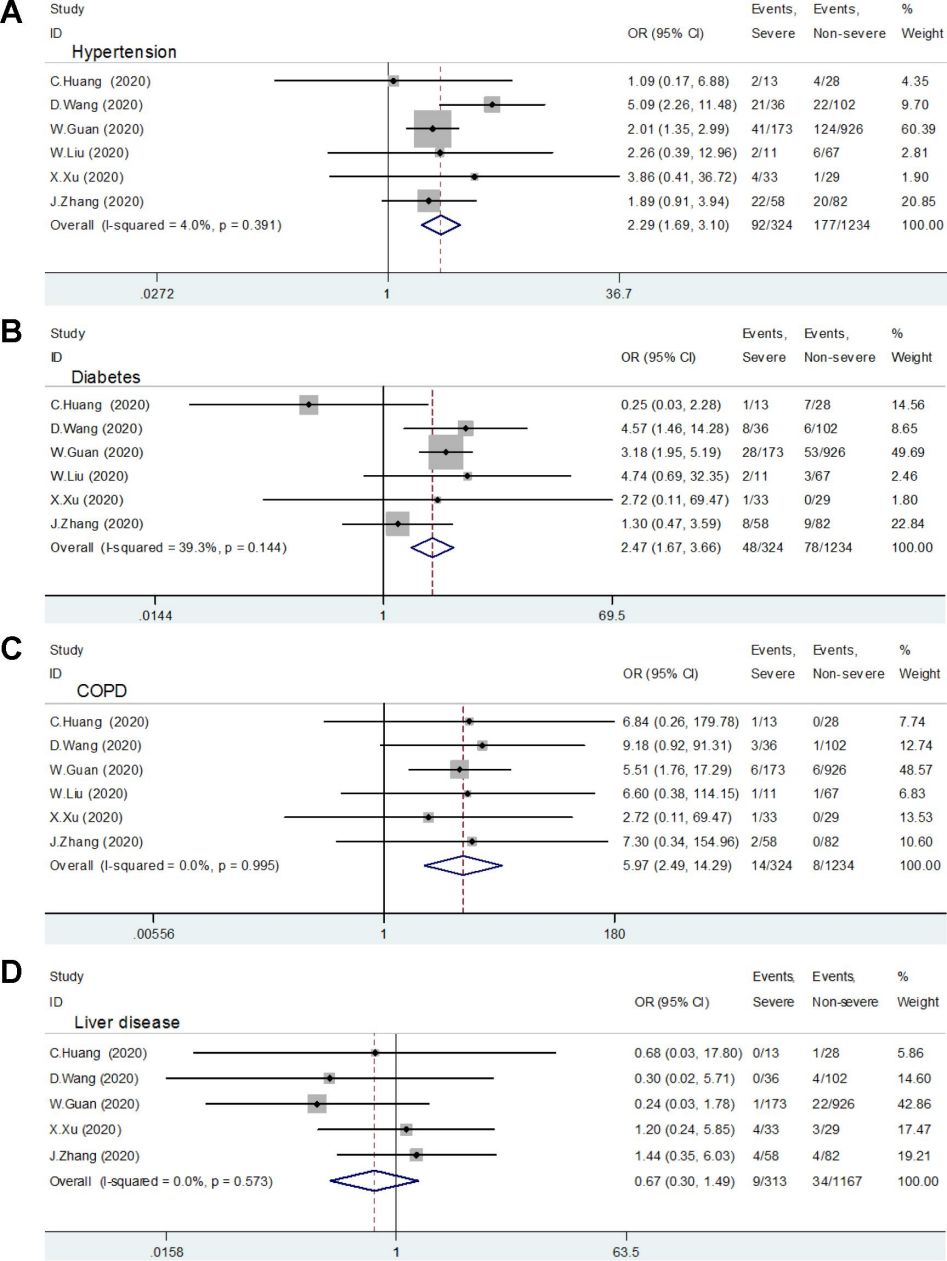
**Relationship between comorbidity and patients with COVID-19.** (**A**) Hypertension; (**B**) Diabetes; (**C**) COPD; (**D**) Liver Disease.

**Figure 2:**
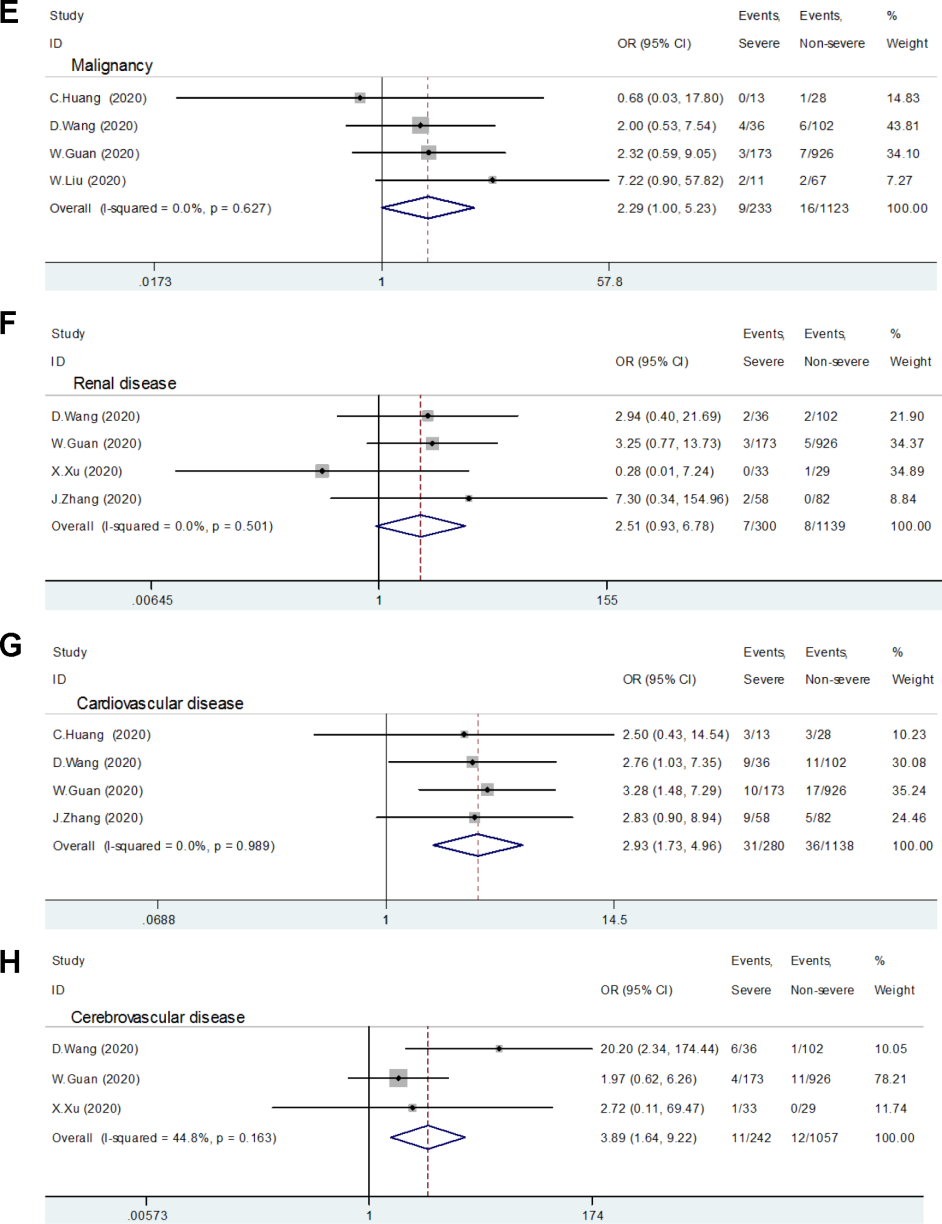
**Relationship between comorbidity and patients with COVID-19.** (**E**) Malignancy; (**F**) Renal disease; (**G**) Cardiovascular disease; (**H**) Cerebrovascular disease.

### Cardiovascular disease and cerebrovascular disease

Four included studies reported the relationship between cardiovascular disease and patients with severe COVID-19 [1, 4–6]. No significant heterogeneity was found (I^2^ =0, P=0.989) among these trials, so a fixed effect pattern was selected. The results showed that cardiovascular disease is a risk factor for patients with COVID-19 (OR:2.93, 95% CI: 1.73-4.96, P<0.001) (Figure 2G).

Three studies provided the data in terms of cerebrovascular disease [4, 5, 7]. A fixed-effects model was used since the heterogeneity test suggested that there was no significant heterogeneity (I^2^ =44.8%, P=0.163). The meta-analysis shows a significant relationship between patients with severe COVID-19 and cerebrovascular disease (OR:3.89, 95% CI: 1.64-9.22, P=0.002) (Figure 2H).

### Liver disease, malignancy, and renal disease

Five studies comprising 313 severe group cases and 1167 non-severe group cases evaluated the role of liver disease in patients with COVID-19 [1, 4–7]. The meta-analysis showed that patients with the previous liver disease did not increase the risk of disease progression (OR:0.67, 95% CI: 0.30-1.49, P=0.326) (Figure 2D).

The relative risk assessments associated with malignancy and kidney disease are presented in Figure 2E and 2F, respectively. The meta-analysis suggested that there was no correlation between malignant tumor (the 95% confidence interval includes 1) or kidney disease (P=0.070) and COVID-19 patients' aggravation.

### Subgroup analysis

To further verify the correlation of comorbidity and COVID-19 patients' aggravation, subgroup analysis was conducted. The results of the subgroup analysis are presented in Table 2. The Subgroup analysis results further support the results of hypertension, COPD, liver disease, and renal disease. In the clinical symptom group, we further observed that hypertension, diabetes, COPD, malignancy, and cardiovascular disease were a risk factor in COVID-19 patients.

**Table 2:** Results of meta-analysis and subgroup analysis.

	**No. of studies**	**OR(95%CI)**	**P-Value**	**Heterogeneity**		**Model used**
				***I²***	***P_h_***	
Hypertension	6	2.29(1.69-3.10)	<0.001	4.0%	0.391	Fixed
ICU care	2	2.97(0.70-12.55)	<0.001	55.7%	0.133	Romdon
Clinical symptoms	4	2.03(1.45-2.85)	<0.001	0	0.947	Fixed
Diabetes	6	2.47(1.67-3.66)	<0.001	39.3%	0.144	Fixed
ICU care	2	1.24(0.07-22.98)	0.883	82.0%	0.018	Romdon
Clinical symptoms	4	2.66(1.73-4.10)	<0.001	0	0.429	Fixed
COPD	6	5.97(2.49-14.29)	<0.001	0	0.995	Fixed
ICU care	2	8.30(1.26-54.43)	0.027	0	0.885	Fixed
Clinical symptoms	4	5.37(1.99-14.46)	0.001	0	0.973	Fixed
Liver disease	5	0.67(0.30-1.49)	0.326	0	0.573	Fixed
ICU care	2	0.41(0.05-3.53)	0.713	0	0.416	Fixed
Clinical symptoms	3	0.74(0.31-1.75)	0.492	16.9%	0.300	Fixed
Malignancy	4	2.29(1.00-5.23)	0.049	0	0.627	Fixed
ICU care	2	1.67(0.49-5.61)	0.410	0	0.547	Fixed
Clinical symptoms	2	3.18(1.05-9.64)	0.041	0	0.370	Fixed
Renal disease	4	2.51(0.93-6.78)	0.070	0	0.501	Fixed
ICU care	1	2.94(0.40-21.69)	0.290	-	-	-
Clinical symptoms	3	2.38(0.76-7.50)	0.237	15.0%	0.308	Fixed
Cardiovascular disease	4	2.93(1.73-4.96)	<0.001	0	0.989	Fixed
ICU care	2	2.69(1.14-6.34)	0.023	0	0.924	Fixed
Clinical symptoms	2	3.10(1.59-6.02)	0.001	0	0.834	Fixed
Cerebrovascular disease	3	3.89(1.64-9.22)	0.002	44.8%	0.163	Fixed
ICU care	1	20.20(2.34-174.44)	0.006	-	-	-
Clinical symptoms	2	2.07(0.70-6.12)	0.189	0	0.852	Fixed

### Publication bias

**Figure 3:**
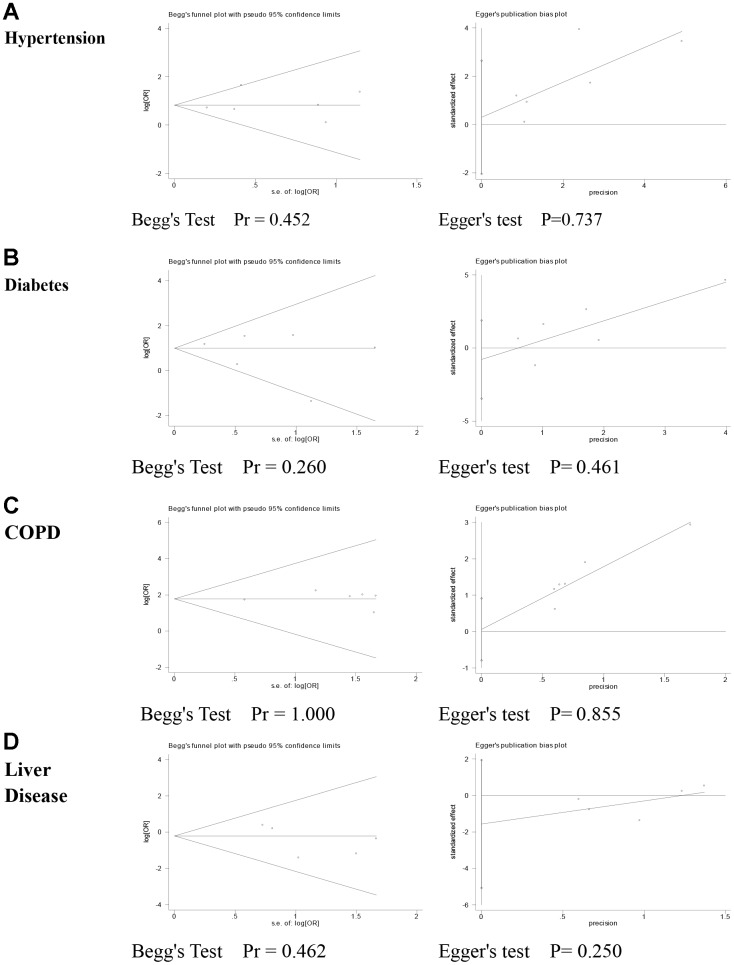
**Publication bias assessment.** (**A**) Hypertension; (**B**) Diabetes; (**C**) COPD; (**D**) Liver Disease.

The risk of publication bias was analyzed in the following comorbidities: hypertension, diabetes, COPD, and liver disease. Figure 3 shows the results of publication bias, which were evaluated by funnel plots and Eggers test. Begg’s test (All Pr>0.05) and Egger’s regression test (All P >0.05) suggest no significant publication bias.

## DISCUSSION

Currently, the increasing number of cases and extensive geographical expansion of the COVID-19 are causing widespread concern in the world [9]. Tian et al [10]. described that the proportion of severe versus common cases of the COVID-19 infection, which was approximately 1:4, the ratio of severe to mild were 18% and 73%. Until now, the source and pathogenesis of the COVID-19 remain unclear, and no specific treatment has been recommended for coronavirus infection except for meticulous supportive care [8, 11]. Unfortunately, in severe patients with COVID-19, the disease progresses rapidly, and respiratory failure can occur within a short time, even leading to death. Early data from Wuhan Jinyintan Hospital showed that 61.1% of patients in ICU had respiratory failure, 44.4% had arrhythmia, and 30.6% had a shock [11]. Therefore, early identification of severe patients is of great significance for improving the therapeutic effect of COVID-19 and reducing mortality.

Previous studies have described that the presence of common comorbidities increase COVID-19 patients’ risk [5]. Besides, some scholars think that the presence of any coexisting illness was more common among patients with severe disease than among those with the non-severe disease [4]. However, the specific comorbidity by which can lead to disease progression remain unknown in COVID-19 patients.

A total of 1558 COVID-19 patients were included in the analyses, 324 (20.8%) of whom were severe. The meta-analysis of retrospective studies confirms that COPD is associated with a dramatically increased risk of aggravation in patients with COVID-19. COVID-19 patients with COPD had a 5.9-fold higher risk of progression than patients without COPD. Moreover, we identify an increased risk of aggravation in individuals who have hypertension, diabetes, cardiovascular disease, or cerebrovascular disease. Our meta-analysis did not provide sufficient evidence that there was a correlation between liver disease, malignant tumor or kidney disease, and COVID-19 patients' aggravation.

However, this conclusion needs to be taken with caution, as this study has several limitations. Firstly, the small sample size may reduce the significance of the results. Secondly, the judgment criteria for severe and non-severe patients included in the study were not uniform. Thirdly, some included patients who had more than one coexisting illness. Fourth, the quality of different studies was different, which might lead to bias.

## CONCLUSIONS

The meta-analysis identified hypertension, diabetes, COPD, cardiovascular disease, and cerebrovascular disease as significant risk factors for COVID-19 patients. The knowledge of these factors can better define those COVID-19 patients at higher risk, and thus allow a more targeted and specific approach to prevent those deaths. Given the limitations of this conclusion, well-designed trials of high quality are needed to explore the relationship between comorbidity and patients with COVID-19.

## MATERIALS AND METHODS

### Search strategy and study selection

The Meta-analysis was performed according to the Preferred Reporting Items for Systematic reviews and Meta-analysis (PRISMA) statement [12]. Relevant literature was extracted by systematic retrieval of PubMed (Medline), EMBASE, Springer, Web of Science, and Cochrane Library databases up to date to March 1, 2020. Our search strategy included terms for “2019-nCoV” or “Coronavirus” or “COVID-19” or “SARS-CoV-2” or “2019-nCoV” or “Wuhan Coronavirus.” Besides, we manually screened out the relevant potential article in the references selected. The above process was performed independently by two participants.

Inclusion criteria are as follows: (1) Types of Studies: published studies reported the relationship between comorbidity and patients with COVID-19; (2) Subjects: diagnosed patients with COVID-19; (3) Exposure intervention: COVID-19 patients with comorbidity included: hypertension, diabetes, chronic obstructive pulmonary disease (COPD), liver disease, malignancy, renal disease, cardiovascular disease, cerebrovascular disease; (4) Outcome indicator: the odds ratios (OR) with 95% confidence intervals (CI) for each comorbidity.

The exclusion criteria: (1) Case reports, reviews, summaries of discussions, (2) Insufficient data information provided; (3) Patients were not stratified for the degree of severity.

### Data extraction and quality assessment

Two participants separately conducted literature screening, data extraction, and literature quality evaluation, and any differences could be resolved through discussion or a third analyst. Information extracted from the included literature: first author surname, year of publication, country of the population, sample size, relevant data on comorbidity of severe and non-severe patients, etc.

The Newcastle-Ottawa scale (NOS) was adopted to evaluate the process in terms of queue selection, comparability of queues, and evaluation of results [13]. The quality of the included studies was assessed independently by two participants. NOS scores of at least six were considered high-quality literature. Higher NOS scores showed higher literature quality.

### Statistical analysis

All data analysis was performed using Stata12.0 software (Stata Corp, College Station, Texas). The OR and relevant 95% CI were used to estimate pooled results from studies. After that, the heterogeneity test was conducted. When P≥0.05 or I^2^<50% was performed, it indicated that there was no obvious heterogeneity, and the fixed-effect model should be applied for a merger. Otherwise, the random-effect model was applied. Results were considered significant statistically when the p-value less than 0.05.

Studies were grouped according to the type of disease severity judgment basis. One subgroup is based on the clinical symptoms of patients, and the other subgroup is based on whether patients experience ICU care or not. Subgroup sensitivity analyses were conducted to explore potential sources of heterogeneity.

Publication bias was assessed using Begg funnel plot and Egger test linear regression test (where at least five studies were available). If P < 0.05 indicates obvious publication bias.
